# High OXPHOS efficiency in RA-FUdr-differentiated SH-SY5Y cells: involvement of cAMP signalling and respiratory supercomplexes

**DOI:** 10.1038/s41598-024-57613-x

**Published:** 2024-03-28

**Authors:** Maria Laura Matrella, Alessio Valletti, Isabella Gigante, Domenico De Rasmo, Anna Signorile, Silvia Russo, Simona Lobasso, Donatella Lobraico, Michele Dibattista, Consiglia Pacelli, Tiziana Cocco

**Affiliations:** 1 Department of Translational Biomedicine and Neuroscience, University of Bari Aldo Moro, 70124 Bari, Italy; 2 National Institute of Gastroenterology- IRCCS “Saverio De Bellis”, Via Turi 27, Castellana Grotte, 70013 Bari, Italy; 3 Bioenergetics and Molecular Biotechnologies, CNR-Institute of Biomembranes, 70124 Bari, Italy; 4 Department of Clinical and Experimental Medicine, University of Foggia, 71122 Foggia, Italy; 5 MASMEC Biomed S.p.A, 70026 Modugno, Italy

**Keywords:** Biochemistry, Neuroscience

## Abstract

Neurons are highly dependent on mitochondria to meet their bioenergetic needs and understanding the metabolic changes during the differentiation process is crucial in the neurodegeneration context. Several in vitro approaches have been developed to study neuronal differentiation and bioenergetic changes. The human SH-SY5Y cell line is a widely used cellular model and several differentiation protocols have been developed to induce a neuron-like phenotype including retinoic acid (RA) treatment. In this work we obtained a homogeneous functional population of neuron-like cells by a two-step differentiation protocol in which SH-SY5Y cells were treated with RA *plus* the mitotic inhibitor 2-deoxy-5-fluorouridine (FUdr). RA-FUdr treatment induced a neuronal phenotype characterized by increased expression of neuronal markers and electrical properties specific to excitable cells. In addition, the RA-FUdr differentiated cells showed an enrichment of long chain and unsaturated fatty acids (FA) in the acyl chain composition of cardiolipin (CL) and the bioenergetic analysis evidences a high coupled and maximal respiration associated with high mitochondrial ATP levels. Our results suggest that the observed high oxidative phosphorylation (OXPHOS) capacity may be related to the activation of the cyclic adenosine monophosphate (cAMP) pathway and the assembly of respiratory supercomplexes (SCs), highlighting the change in mitochondrial phenotype during neuronal differentiation.

## Introduction

There is increasing evidence that neuronal differentiation, maturation, and aging are closely linked to changes in cellular metabolism, in particular, cellular bioenergetics^1–5^. In vitro neuronal models represent a valuable tool to overcome the problems associated with the use of primary neurons, such as their limited lifespan, which restricts the number of cells available for investigation and the need to obtain ethical approval for protocols. Several in vitro approaches have been developed to advance knowledge in neuroscience and for elucidation of the pathogenic mechanisms of a variety of neurodegenerative diseases. These include the use of immortalized cell lines of neuronal origin^6^, human induced pluripotent stem cells (iPSCs)^7^ and reprogrammed cells from primary cultures^8,9^ in 2D^10^ and 3D cell cultures or organized in organoids^11^. Immortalized cell lines, modified to proliferate indefinitely, are commonly used due to their ability to grow rapidly over a high number of passages and their low cost of purchase and maintenance. Despite a number of limitations that need to be taken into account when using the SH-SY5Y neuroblastoma cell line, e.g. the presence of two morphologically distinct phenotypes^12^, the lack of clearly defined neuronal subtypes^13^ and large-scale chromosomal rearrangements^14^ (see also^15^) they are widely used as an in vitro model in neuroscience research.

Specifically, due to its human origin, catecholaminergic neuronal properties, and ease of handling, the SH-SY5Y have been most suitable for studying neurodegeneration^16^, neurotoxicity^17^ and neurite outgrowth processes^18,19^. Although SH-SY5Y cells are often used in an undifferentiated state^20,21^, differentiation is required to obtain a cell population whose morphological and functional behavior resembles that of mature neurons, including the extension of neurite-like processes, the expression of specific neuronal markers and receptors and the ability to fire action potentials^22–24^. Several differentiation protocols have been developed to induce neuron-like phenotypes in SH-SY5Y cells using specific agents such as phorbol esters, brain-derived neurotrophic factor (BDNF), nerve growth factor (NGF) and B-27 supplement^19,25–27^.

The most widely used method is the treatment with retinoic acid (RA) which has been reported to promote the expansion of neuritic processes, the expression of neuron-specific markers such as microtubule-associated protein 2 and beta III tubulin^28^ and the transition from a proliferating neuroblastoma phenotype to post-mitotic differentiated cells with morphological and functional properties associated with mature neurons, such as the ability to generate free action potentials^29^. During treatment with RA, some epithelial-like cells appear to be unaffected by the effects of this agent thus resulting in a mixed cell population characterized by an increased number of undifferentiated SH-SY5Y cells that continue to proliferate, therefore leading to uncertain results^30^. The use of RA in combination with exogenous trophic factors such as brain-derived neurotrophic factor (BDNF) has been reported to produce a fully differentiated population of neuron-like cells^25,31^. Although this neurotrophin is widely used, cell cycle inhibition using this protocol is not reliable^22,32^. Furthermore, neurotrophins such as nerve growth factor (NGF) or BNDF activate a PI3Kinase/ Akt signalling pathway to promote cell survival, limiting the evaluation of the effects of exogenous neuroprotective agents^33,34^.

A major issue associated with the retinoic acid differentiation approach and with other differentiating agents is the purity of the resulting cell culture^30^. To get a homogeneous cell culture of differentiated SH-SY5Y cells without the need for trophic support, here we used a two-step differentiation method combining RA treatment with FUdr, essentially as described in previous studies^35,36^.

Specifically, FUdr is an inhibitor of thymidylate synthase (TS) which causes an imbalance in the intracellular deoxyribonucleoside triphosphate pool for DNA replication^37^ and, at low doses, affects dividing proliferating cells and does not affect post-mitotic not-dividing brain cells.

RA-mediated in vitro neuronal differentiation is known to be associated with increased mitochondrial respiratory capacity^18,38,39^ and the upregulation of ATP production pathways to support axon and dendrite extension and synapse formation^40^. In this work we functionally and metabolically characterized the RA-FUdr differentiated neuronal cells. Specifically, we showed that RA-FUdr treatment induced differentiation in neuronal-like cells characterized by a mitochondrial energetic phenotype supported by an increase in the assembly of respiratory SCs. In addition, our results suggest that the observed metabolic changes in RA-FUdr differentiated neuronal cells may be related to the activation of cAMP pathway.

## Results

### RA-FUdr treated SH-SY5Y displays a neuronal phenotype

**Figure 1:**
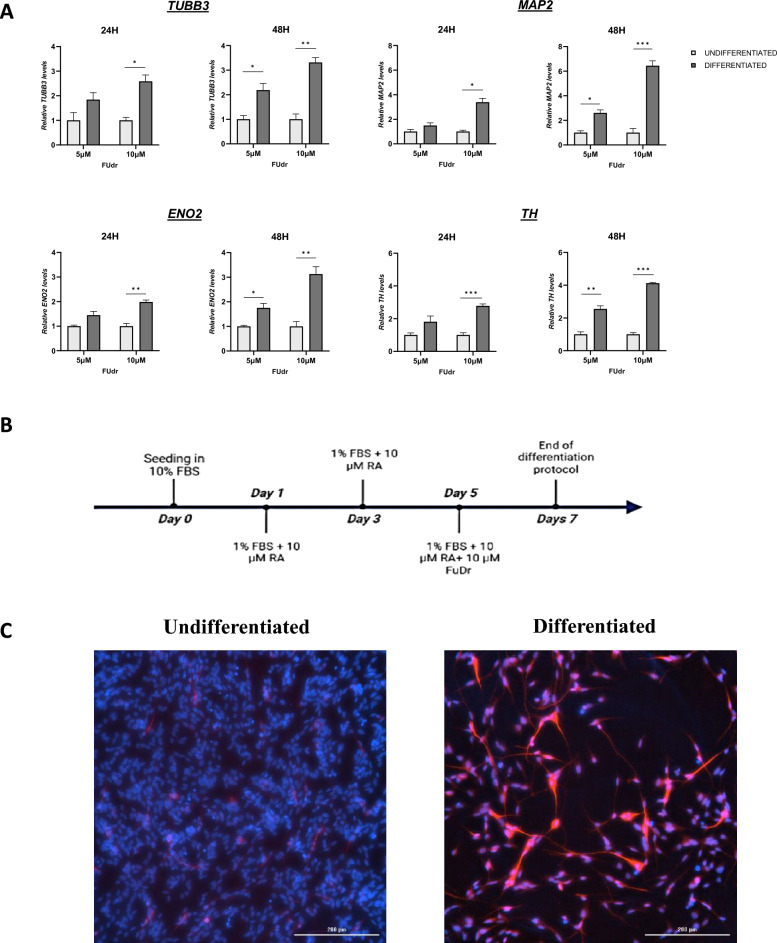
Phenotypic characterization of RA-FUdr-differentiated SH-SY5Y cells. (**A**) Transcript levels of TUBB3, MAP2, ENO2 and TH were measured in SH-SY5Y cells treated with 10 µM RA in combination with 5 or 10 µM FUdr for the last 24 h and 48 h of treatment. Relative mRNA levels were evaluated by qRT-PCR and normalized to the housekeeping gene *GAPDH*. Data, expressed as fold-change mRNA expression levels in differentiated cells, compared to undifferentiated cells, are means ± SEM of two replicates from three independent experiments. Statistical analyses were performed using unpaired t-test with Welch’s correction (*p < 0.05; **p < 0.01; ***p < 0.001 Student’s t-test). (**B**) Timeline of the experimental setup for the cell differentiation protocol. Cells were cultured for 7 days in a culture medium supplemented with 10 µM RA supplemented with 10 µM FUdr in the last two days of treatment (**C**) Representative images showing immunofluorescence staining for MAP2 in undifferentiated and differentiated cells. Images were acquired using the Cytation 5 Cell Imaging Multi-Mode Reader (BioTek) with a 20 × objective lens (scale bar = 200 µm). MAP2 images were acquired using the Texas red filter (excitation 586 nm; emission 647 nm), while DAPI nuclei images were acquired using the DAPI filter (excitation 377 nm; emission 447 nm).

To achieve greater neuronal differentiation without issues arising from a mixed cell population, we have utilized a two-step method combining treatment with RA and FUdr essentially as previously described^35,36^. The SH-SY5Y cells were cultured for seven days in the presence of 10 µM RA, serum reduction (1%) supplemented with FUdr in the last two days of treatment, while the undifferentiated cells were treated with dimethyl sulfoxide (DMSO, vehicle) in a low serum medium for seven days, supplemented with FUdr in the same condition of RA-treatment. The appropriate FUdr concentrations and exposure times to obtain an enriched neuron-like cell population were selected on the basis of preliminary experiments. We treated the SH-SY5Y cells with RA for seven days supplemented with 5 µM or 10 µM FUdr in the last 24 h and 48 h of treatment and measured the mRNA levels of neuronal markers the day after the seventh day of treatment. For SH-SY5Y differentiation, cells were seeded and cultured in fresh media supplemented with 1% FBS after 48 h of plating. In Fig. 1A the experimental groups were as follows: undifferentiated cells treated with DMSO for 5 days, supplemented with 5 µM FUdr for the last 24 h and 48 h of treatment; undifferentiated cells treated with DMSO for 5 days, supplemented with 10 µM FUdr for the last 24 h and 48 h of treatment; RA-differentiated cells treated with 10 µM RA for 5 days, supplemented with 5 µM FUdr for the last 24 h and 48 h of treatment; differentiated cells treated with 10 µM RA for 5 days, supplemented with 10 µM FUdr for the last 24 h and 48 h of treatment. The day after the seventh day of treatment, cells were harvested for qRT-PCR analysis. The highest expression of neuronal markers was observed in SH-SY5Y cells treated with RA for seven days supplemented with 10 µM FUdr for the last 48 h (Fig. 1A). In this condition, tubulin beta-3 class III (*TUBB3*), neuron-specific enolase (*ENO2*), as well as tyrosine hydroxylase (*TH*), a dopaminergic neuronal marker showed an approximately threefold increase whereas the microtubule-associated protein-2 (*MAP2*) and an approximately sixfold increase compared with undifferentiated cells.

These results suggest that prolonged incubation with a higher concentration of FUdr improves the homogeneity of the culture, probably by reducing the number of non-neuronal cells and based on these results, this two-step RA-FUdr differentiation protocol was the condition used for all further experiments as described in the schematic diagram of the culture protocol in Fig. 1B.

At the end of the differentiation treatment, we confirmed the establishment of a neuronal phenotype by determining the distributional pattern of MAP2-like immunoreactivity (Fig. 1C). FUdr treatment inhibits SH-SY5Y cell proliferation and the RA-FUdr treatment induces a post-mitotic state of the cells, which exhibited dendrite-like processes and a network formation in the whole cell population.

Specifically, RA-FUdr differentiated cells showed significant neurite outgrowth with intense staining for MAP2 compared to undifferentiated cells. Furthermore, analysis of images stained for MAP2 showed that all differentiated cells in our cultures expressed this neuronal marker, suggesting the homogeneity of the resulting neuronal-like population.

### RA-FUdr treated SH-SY5Y display neuron-like voltage gated currents

The functional screening revealed that, in addition to the aforementioned markers, the emergence of voltage-gated currents characterizes the differentiation of SH-SY5Y cells into neuron-like cells. We used the patch-clamp technique to investigate their electrophysiological properties.

First, we observed that the resting membrane potential of differentiated cells (Vrest = − 61.50 mV, Table 1) was significantly more negative compared with undifferentiated cells (Vrest = − 44.63 mV, Table 1). However, no differences in input resistance were detected, suggesting that a main feature of RA-differentiated SH-SY5Y cells exhibiting a neuron-like phenotype is their more negative resting membrane potential.

**Table 1:** Resting membrane potential and input resistance in differentiated and undifferentiated cells.

	Differentiated cells (mean ± SEM, [n])	Undifferentiated cells (mean ± SEM, [n])	p-value
Passive properties			
Resting membrane potential (mV)*	− 61.50 ± 2.09, [14]	− 44.63 ± 3.76, [8]	< 0.001
Input resistance (Ω)	5.25 ± 0.86, [14]	4.685 ± 1.21, [8]	0.69

Data are presented as the mean ± SEM values of passive properties of differentiated and undifferentiated cells. Statical differences between groups were determined with one-way ANOVA, followed by Tukey test as post-hoc analysis.

*Statistically significant difference.

**Figure 2:**
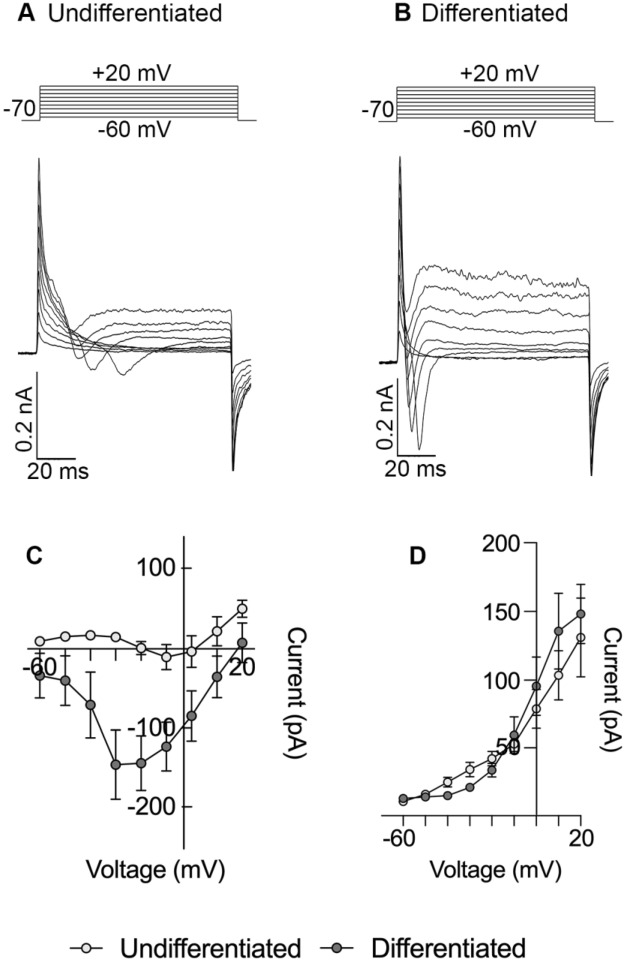
Electrophysiological current responses in undifferentiated and RA-FUdr-differentiated cells. Representative whole-cell current traces recorded from undifferentiated (**A**) and differentiated (**B**) cells using the voltage protocols indicated at the top of the panels. Depolarizing voltage steps were applied with an increment of 10 mV each for 100 ms, starting from − 60 mV, holding the cells at − 70 mV. Plot of average ± SEM amplitudes of inward (**C**) and outward (**D**) currents. Unpaired Mann–Whitney with the Benjamini–Hochberg (BH) method n = 11 (differentiated), n = 7 (undifferentiated).

In the voltage-clamp configuration, we elicited voltage-gated currents by applying voltage steps ranging from − 60 mV to + 20 mV, holding the cells to − 70 mV. In the differentiated cells, the inward currents are activated from a potential of approximately − 40 mV and peaked at − 20 mV (Fig. 2B,C). Inward currents were either diminished or absent in the same voltage range in undifferentiated cells (Fig. 2A,C). The fast kinetics of the currents, along with the I-V relationship, suggest a possible mix of voltage-gated sodium and calcium currents. Furthermore, depolarizing voltage steps also induced outward currents, likely representing outward potassium currents, that developed slowly and reached a steady state at around 100 ms (Fig. 2A,B). There appeared to be no significant difference in these currents between differentiated and undifferentiated cells (Fig. 2D).

### Identification of phospholipid changes in RA-FUdr treated SH-SY5Y cells

Neuronal differentiation, characterized by neurite outgrowth, is critically dependent on membrane biosynthesis and lipid composition changes. To identify potential lipid biomarkers in the cellular RA differentiation process, we compared lipid profiles obtained from undifferentiated and differentiated cells by MALDI-TOF/MS analysis. We acquired the MALDI mass spectra of the total lipid extracts in both negative and positive ionization modes to identify the main lipid species that ionize better in negative (i.e., acidic lipid classes) or positive (i.e., zwitterionic glycerophospholipids) ion mode, depending on their chemical structure. Typical MALDI lipid profiles of cells are shown in Fig. S1, while the main signals associated with the negative [M−H]^−^ and positive [M + H]^+^ molecular ions detected in the mass spectra are summarized in Table S1.

Although the MALDI lipid profiles of the undifferentiated and differentiated cells exhibited many similarities and no qualitative changes have been found between the two lipid patterns (see Fig. S1A,B), we found significant differences in the intensity of MALDI peaks corresponding to some phospholipids, including plasmalogen (Pls) and cardiolipin (CL) species.

**Figure 3:**
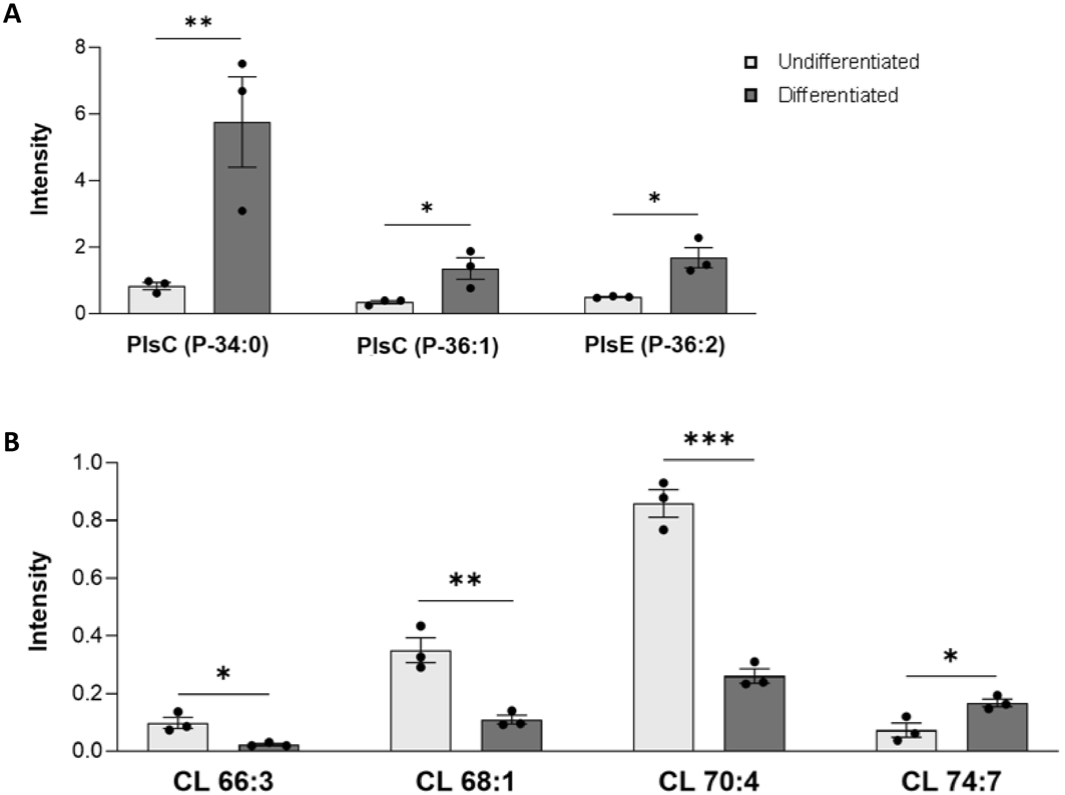
Significant differences in MALDI signals in the lipid profile of undifferentiated and RA-FUdr-differentiated cells. The histograms show the significant differences in intensity between the lipid peaks in the two series of mass spectra. In (**A**) significant changes in plasmalogens (Pls) species [PlsC detected in (+) MALDI mass spectra, PlsE detected in (−) MALDI mass spectra]. In (**B**) significant changes in cardiolipin (CL) species, detected in (−) MALDI mass spectra. Data are expressed as mean of signal intensity ± SEM (n = 3) (*p < 0.05; **p < 0.01; ***p < 0.001). Lipid assignments for each MALDI signal are indicated, as in Table S1.

Specifically, the following MALDI signals corresponding to different Pls species were significantly higher in differentiated cells than in undifferentiated cells: two peaks at *m/z* 746.6 and 772.5, assigned to different plasmenyl-PC species (PlsC P-34:0 and PlsC P-36:1, respectively) and a peak at *m/z* 726.5 assigned to one plasmenyl-PE species (PlsE P:36:2) (Fig. 3A). These results suggest an enrichment of PLs species in the membranes of differentiated cells.

Regarding CL, the key mitochondrial phospholipid, MALDI-TOF/MS analysis of lipids revealed the presence of several species in both undifferentiated and differentiated cells (CL 66:3, CL 68:1, CL 70:4, CL 72:4 and CL 74:7 with peaks at *m/z* 1374.1, 1402.1, 1428.1, 1456.1, and 1478.2, respectively) (Fig. S1C). Interestingly, comparative analysis revealed a significant change in CL-acyl composition between undifferentiated and differentiated cells. Differentiated cells showed lower levels of CL species with shorter and more saturated fatty acyl-chains (CL 66:3, CL 68:1, CL 70:4) and higher levels of CL species with longer fatty acyl-chains and higher degree of unsaturation (CL74:7) compared to undifferentiated cells (Fig. 3B). These results are consistent with the CL profile of brain tissue which shows a high diversification and enrichment of long-chain polyunsaturated CL species^41^.

In general, during differentiation, neurons require a large amount of lipids for membrane formation in the dendritic growth process^42^. Previous studies reported that activation of lipid metabolism occurred in RA-treated cells^43^. Lipid homeostasis in vertebrate cells is regulated by a family of membrane-bound transcription factors, the sterol regulatory element-binding proteins (SREBPs), which directly promote the expression of lipogenic genes such as acetyl-CoA synthase (acs), acetyl-CoA carboxylase (*ACC*) and fatty acid synthase (*FASN*). SREBP-1C is the major isoform found in most tissues, including brain, and regulates FA and triglyceride synthesis^42^. Here we observed significantly increased expression of *SREBP-1C* (eightfold increase) and *FASN* (twofold increase) in SH- SY5Y differentiated cells (Fig. S2), which may promote lipid synthesis required for neurogenesis and neurite outgrowth processes.

### RA-FUdr-induced SH-SY5Y differentiation enhances the bioenergetic metabolism

**Figure 4:**
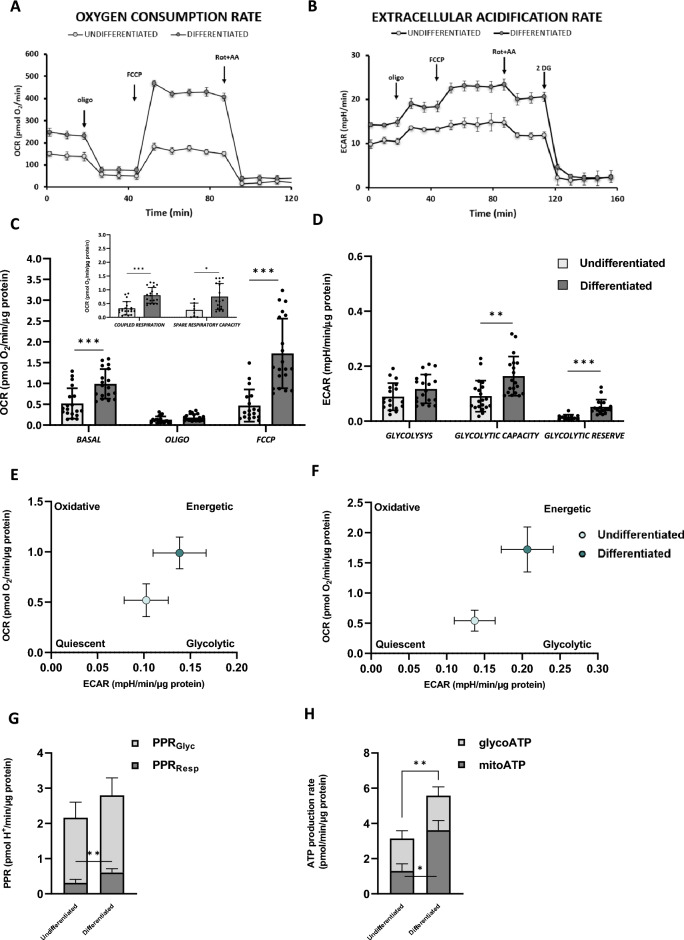
Oxidative phosphorylation activation in RA-FUdr-differentiated SH-SY5Y cells. Cells were seeded in 24-well microplates and bioenergetic function was assessed using the Seahorse XF24 Analyzer. Representative profile of Oxygen Consumption Rate (OCR) (**A**) and Extracellular Acidification Rate (ECAR) (**B**) of undifferentiated and differentiated SH-SY5Y cells, recorded at baseline and after injection of ATP synthase inhibitor oligomycin (oligo), uncoupler FCCP, complex I inhibitor rotenone (Rot) plus complex III inhibitor antimycin A (AA) and 2-deoxy glucose (2DG). Each data point is the mean ± SD of five technical replicates. (**C**, **D**) Metabolic parameters obtained from OCR and ECAR assays, respectively. Data are the mean ± SEM of 4 independent experiments with five technical replicates each and refer to the Rot + AA-sensitive OCR and 2DG-sensitive ECAR normalized to the protein levels. (**C**) Bar graphs of basal OCR (BASAL) and OCR in the presence of oligomycin (OLIGO) and FCCP (FCCP); in inset, quantitative analysis of OCR expressed as pmol/min/µg protein, in undifferentiated and differentiated cells defined for Coupled Respiration and Spare Respiratory Capacity. (**D**) Quantitative analysis of ECAR expressed as mpH/min/µg protein, in undifferentiated and differentiated cells derived for Glycolysis, Glycolytic Capacity, and Glycolytic Reserve. See “Methods” for experimental details. Statistical analyses were performed using unpaired t-test with Welch’s correction (*p < 0.05, **p < 0.01, ***p < 0.001 Student’s t-test). (**E**, **F**) Energy maps for undifferentiated and differentiated cells were obtained by plotting OCR *versus* ECAR values under basal (**E**) and maximal (after FCCP injection) conditions (**F**). (**G**) Proton production rates (PPR) from glycolytic lactate (glycoPPR) and respiratory CO_2_ production (mitoPPR) in undifferentiated and differentiated cells were expressed as pmol H^+^/min/µg protein. All data are mean ± SEM of n = 4 biological replicates. (**p < 0.01 Student’s t-test) (**H**) ATP production rate expressed as pmol ATP/min/µg protein. MitoATP and GlycoATP are shown as stacked bars and are the means ± SEM of 4 independent experiments. Statistical analyses were performed using unpaired t-test with Welch’s correction (*p < 0.05 and **p < 0.01).

Given the important role of mitochondria in the energy profile in neuronal differentiation^1,2,44–46^ and the determinant role of CL acyl chains composition for mitochondrial function and energy metabolism^41,47,48^, we examined whether RA-FUdr treatment affected mitochondrial bioenergetics. To this aim, we performed a systematic analysis using the seahorse technology to assess the two main metabolic fluxes in intact cells: (i) mitochondrial OXPHOS activity, measured as oxygen consumption rate (OCR); and (ii) extracellular acidification rate (ECAR) mainly contributed by the conversion of pyruvate to lactate, essentially according to the manufacturer’s Mito Stress protocol^49^. Figure 4 shows the representative OCR (A) and ECAR (B) profiles in differentiated and undifferentiated cells. The bar graphs show the quantification of the data obtained by comparing differentiated and undifferentiated cells OCR and ECAR activities corrected for non-mitochondrial respiration, after the addition of the mitochondrial respiratory chain inhibitors rotenone plus antimycin A for OCR (Fig. 4C) and the glycolysis inhibitor 2-deoxyglucose for ECAR (Fig. 4D). Differentiated cells showed a significantly higher OCR activity compared to undifferentiated cells under basal conditions (Fig. 4C), the minimum oxygen respiration rate required to maintain cell function, suggesting a preference for OXPHOS to meet energy demands under these conditions. After addition of oligomycin, the H^+^-ATP synthase inhibitor, OCR was decreased to similar levels in differentiated and undifferentiated cells (Fig. 4C), indicating that there were no differences in the membrane proton leak. Addition of the uncoupler FCCP stimulated OCR to its maximal activity in both differentiated and undifferentiated cells (Fig. 4C). However, whereas in differentiated cells there is an increase of approximately 70% compared with baseline, the undifferentiated cells, were very close to their maximal activity under basal conditions. As a result, both OCR linked to ATP synthesis (i.e., the difference between basal OCR and OCR plus oligomycin) and the spare respiratory capacity (SRC) (i.e., the difference between OCR plus FCCP and basal OCR), were significantly higher (245%) and (284%) respectively, in differentiated cells compared to undifferentiated cells (see inset Fig. 4C).

To sum up, in our conditions, RA-FUdr differentiated cells exhibited a bioenergetic metabolism mainly based on OXPHOS activity and a high mitochondrial reserve capacity necessary to meet additional energy demand (high adaptability to stress conditions).

The Mito Stress protocol provides information, moreover, on Extracellular Acidification Rates (ECAR), mostly a measure of glycolytic flux. ECAR activities, with parameter values corrected for residual activity in the presence of the glycolysis inhibitor 2DG, are displayed in Fig. 4D. Basal glycolysis (the difference between basal and *plus*-2DG ECAR) in differentiated cells was comparable to that observed in undifferentiated cells. In contrast, a significant increase in glycolytic capacity (the difference between *plus*-FCCP ECAR and *plus*-2DG ECAR) (Fig. 4D), was observed under conditions of maximal stimulation of glycolysis achieved by FCCP treatment (Fig. 4D). Moreover, the glycolytic reserve (the difference between *plus*-FCCP and basal ECAR) was higher in differentiated cells than in undifferentiated cells (Fig. 4D).

When we analysed the bioenergetic profiles obtained from the resting extracellular acidification rate and oxygen consumption rate values from the data shown in Fig. 4C and D, a different pattern emerged: whereas the undifferentiated cells were restricted to the low-energy-glycolytic region of the bioenergetic profile, differentiated cells showed a more energetic phenotype, both under basal (Fig. 4E) and maximal conditions (Fig. 4F), indicating a functional metabolic reprogramming based on increased mitochondrial OXPHOS and glycolytic capacity to support high cellular activity^5^.

As the overall extracellular acidification is a result of glycolytic lactate production and oxidative reactions, we derived the rate of ATP production using a recently developed and validated protocol based on seahorse technology. By comparing the rates of O_2 _consumption and extracellular H^+^ release, measured simultaneously on the same sample, it is possible to determine the rate of ATP produced by both OXPHOS (mitoATP) and glycolysis (glycoATP) as previously described^50–53^. A clear advantage of this method is the ability to directly quantify changes in the balance between oxidative phosphorylation and glycolysis. Under basal conditions, we observed a twofold increase in the proton production rates associated with bicarbonate formed by CO_2_ production (mitoPPR) in differentiated cells compared with undifferentiated cells (Fig. 4G) and, in addition, a threefold increase of mitochondrial ATP production rate (mitoATP) with a switch to OXPHOS as the main ATP generating pathway (Fig. 4H).

### RA-FUdr-induced SH-SY5Y differentiation is associated with mitochondrial biogenesis/turnover

**Figure 5:**
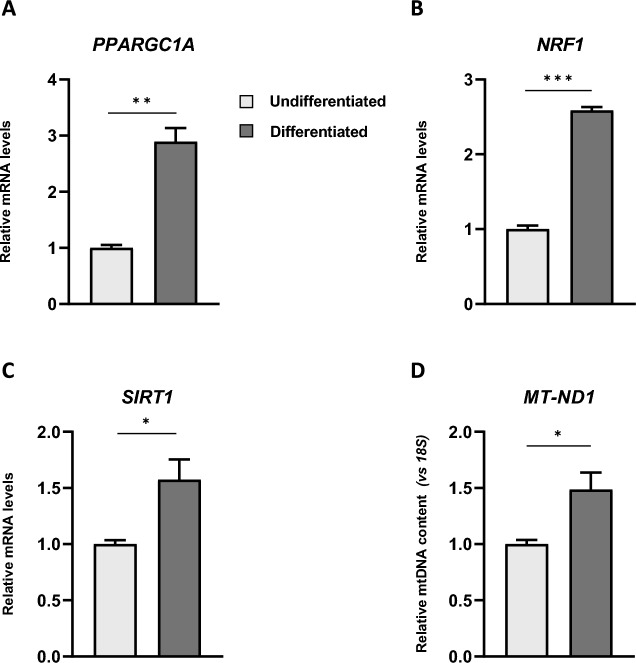
Mitochondrial transcription factors and mitochondrial DNA levels change in RA-FUdr-differentiated SH-SY5Y cells. Relative mRNA levels of *PPARGC1A* (*PGC-1α)* (**A**), *NRF1* (**B**), and *SIRT1* (**C**) in differentiated and undifferentiated cells. The mRNA levels were determined by RT-qPCR of total RNA and normalized to *GAPDH*. Data, expressed as fold-change mRNA expression levels in differentiated cells compared with undifferentiated cells, are means of two replicates ± SEM from three independent experiments. Statistical analyses were performed using unpaired t-test with Welch’s correction (*p < 0.05, **p < 0.01 and ***p < 0.001). See “Methods” for experimental details. (**D**) mtDNA content in differentiated and undifferentiated cells. mtDNA was determined by RT-qPCR amplification of *MT-ND1* normalized to *18S* nuclear DNA. Relative levels of mtDNA/nDNA ratio were compared with undifferentiated cells. Bars are mean ± SEM of three independent experiments, each with two technical replicates. (*p < 0.05 Student’s t-test).

To support our findings on the role of the RA-FUdr-induced differentiation process in improving energy metabolism and to evaluate the molecular mechanisms responsible for the increased reserve capacity, we analysed the expression levels of key factors that regulate mitochondrial biogenesis and function. As shown in Fig. 5, mRNA levels of *PPARGC1A*, *NRF1*, and *SIRT1* were upregulated in differentiated cells compared with undifferentiated cells (Fig. 5A,B,C). At the same time, mitochondrial DNA levels were significantly higher, as shown by the mitochondrial DNA (mtDNA)/nuclear DNA ratio (Fig. 5D). To assess the effect of these key factors on mitochondrial biogenesis, we examined by semi-quantitative western blot analysis the steady-state levels of respiratory chain complex proteins in cell lysates from undifferentiated and differentiated cells. Western blot analysis showed no change in the protein level of 39 kDa, complex I subunit (NDUFA9) and of Core II, complex III subunit (Fig. S4) according to^39^. Regarding the discrepancy with the mRNA expression data (Fig. 5), it is worth remembering that proteins are subject to a cellular physiological turnover, depending on both their synthesis and degradation rates, this could explain the steady-state abundance of proteins analysed.

### cAMP-dependent respiratory SC organization in RA-FUdr-induced SH-SY5Y differentiation

**Figure 6:**
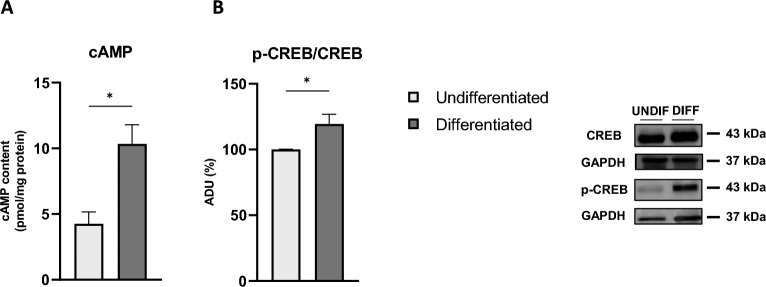
Increased cAMP cellular levels and CREB phosphorylation status in RA-FUdr-differentiated SH-SY5Y cells. (**A**) Basal cAMP levels in total cell lysates expressed as pmol/mg protein in differentiated and undifferentiated cells. Data are mean ± SEM of five independent experiments (*p < 0.05 Student’s t-test). (**B**) Representative Western blot of CREB and p-CREB performed on total cell lysates in differentiated and undifferentiated cells. Bar graph shows p-CREB/CREB ratio determined by quantification using densitometric analysis of band intensity normalized to GAPDH, as loading control. Data are mean ± SEM of five independent experiments, expressed as percentage of undifferentiated cells (*p < 0.05 Student’s t-test).

The cAMP/PKA pathway has an important role in the regulation of mitochondrial structure and function^54–56^. Analysis of total cAMP showed an increase in basal cAMP levels in differentiated cells compared with undifferentiated cells (Fig. 6A), which was associated with an increase in the cAMP-response element-binding protein (CREB) phosphorylation state and, consequently, in the P-CREB/CREB ratio (Fig. 6B). These data suggest the activation of cAMP/PKA pathways in differentiated cells.

**Figure 7:**
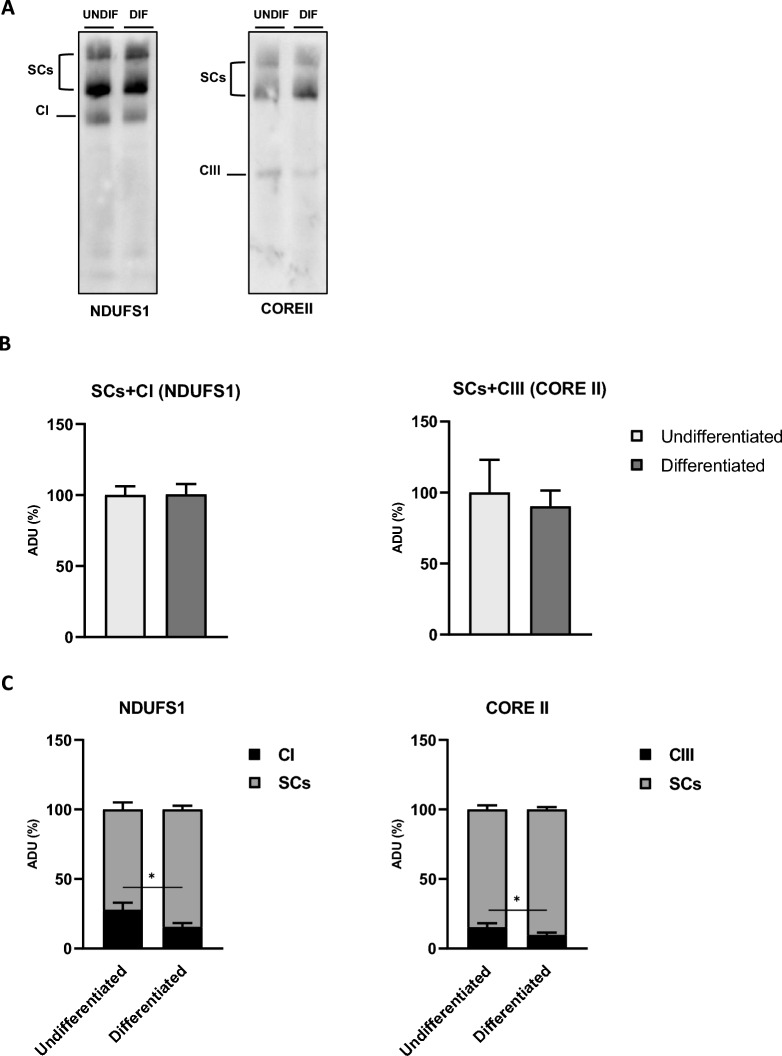
Respiratory SCs reorganization in RA-FUdr-differentiated SH-SY5Y cells. Respiratory chain complexes and SCs from differentiated and undifferentiated cells were prepared as described in “Methods” and separated by 1D-BNE PAGE followed by immunoblotting. (**A**) Representative immunoblotting images showing antibodies against the NDUFS1 subunit of complex I and the Core II subunit of complex III. (**B**) Bar graphs show the quantification of immunoreacted bands as the sum of free complexes (C) and supercomplexes (SCs) expressed as a percentage of arbitrary densitometric unit (ADU). (**C**) Bar graphs show the quantification of bands related to free complexes and SCs in each lane, expressed as a percentage of ADU. See “Methods” for experimental details. Values are mean ± SEM of three independent experiments. Statistical analyses were performed using unpaired t-test (*p < 0.05 Student’s t-test).

Recently it has been described that the activation of the cAMP/PKA cascade results in enhanced formation of respiratory SCs, associated with increased electron flux capacity and ATP production rate^57^. Therefore, the activation of cAMP/PKA signalling and the increase in mitochondrial capacity in differentiated cells prompted to investigate the organization of respiratory chain complexes into SCs. Figure 7 shows the results of blue native polyacrylamide gel electrophoresis (BN-PAGE) of digitonin solubilized cells, followed by immunoblotting analysis with specific antibodies against a subunit of complex I (CI), NDUFS1, and a subunit of complex III (CIII), Core II (Fig. 7A). Western blotting assay with the NDUFS1 antibody revealed a free amount of complex I and a quote of complex I assembled into SCs in differentiated and undifferentiated cells (Fig. 7A). The same has been observed with the antibody against the Core II that revealed free complex III and higher molecular weight bands with the same molecular weight as the bands immuno-revealed with the NDUFS1 antibody (Fig. 7A). These data indicate the presence of SCs containing at least complex I and complex III (Fig. 7A). Densitometric analysis of the bands detected with the NDUFS1 antibody showed that the total amount of complex I (free complex I plus complex I in SCs) did not change in differentiated cells compared with undifferentiated cells (Fig. 7B). Comparable results were obtained by densitometric analysis of immunoblotting with antibody against Core II which showed no change in the total amount of complex III (free complex III plus complex III in SCs) in differentiated cells compared with undifferentiated cells (Fig. 7B).

Although no change was observed for the total amount of complexes I and III, their distribution between free and SCs was affected (Fig. 7C). Indeed, in differentiated cells, both free complex I and complex III decreased, whereas their amount in SCs increased (Fig. 7C).

## Discussion

Human neuroblastoma SH-SY5Y cells are a widely used in vitro cellular model but, although this cell line exhibits many biochemical and functional characteristics of neurons, differentiation is a necessary step to obtain cells expressing mature neuronal markers^39^. Specifically, SH-SY5Y cells can be differentiated in vitro into a dopaminergic phenotype by several methods, including treatment with RA^22,23,28,58–60^. However, prolonged SH-SY5Y RA treatment (7 days) results in the formation of a heterogeneous culture with a mixed population of differentiated and undifferentiated cells. In this study, we used a two-step differentiation protocol to obtain a homogeneous population of cells with neuron-like morphology. Specifically, we induced SH-SY5Y differentiation by treating them with RA for 7 days, in a low-serum cell culture medium supplemented, in the last 2 days, with the drug FUdr, which inhibits the proliferation of undifferentiated cells. In this condition, we obtained cells with neuronal morphological, biochemical, and electrophysiological properties that better resemble mature neurons. The differentiation protocol used results in the induction or increased expression of several neuronal markers, such as TUBB3 and MAP2, protein components of cytoskeleton that are highly expressed in mature neurons, ENO2, an enzyme found in mature neurons and cells of neuronal origin, and TH, the rate-limiting enzyme of catecholamine synthesis according with^58,61^. Although these markers can also be detected in the undifferentiated SH-SY5Y cells, a significant increase in their expression confirms the improvement of differentiation process. During the differentiation process, the SH- SY5Y cells express ion channels^31^ that allow them to acquire a neuron-like phenotype. Therefore, we performed whole-cell recordings to fully understand our homogeneous functional population of neuron-like cells. A first hallmark of the acquired neuron-like phenotype could be the negative shift of the resting membrane potential, which would be crucial to establishing the proper electrochemical gradient for the operation of several voltage-gated ion channels. In addition, the voltage-clamp configuration measured both sodium and calcium currents which are involved in the initiation and propagation of action potentials. These results further strengthen the hypothesis that our protocol promotes the neuron-like electrophysiological maturation of the differentiated SH-SY5Y cells according to other reports^62,63^.

The morphological changes that occur during neuronal differentiation involve additional lipid production for increased membrane requirements. Among the major functions of lipids in the nervous system, their structural role in biological membranes and their contribution to energy supply are of undeniable importance^64^.

Our results revealed important changes in cellular lipidome of the RA- FUdr differentiated SH-SY5Y. These cells contain higher levels of some Pls species (including PlsC and PlsE) compared to undifferentiated cells, and these lipid changes are consistent with the crucial role of Pls in neuronal tissue. The membrane fluidity of neuronal cells, which is correlated with phospholipids containing polyunsaturated acyl chains and, in particular, with Pls, is important for specific functions, including vesicle fusion, neurotransmitter release, and synapse formation^65^. Pls represent a subclass of glycerophospholipids present in mammalian membranes, that contain a vinyl-ether (alkenyl) linkage at the *sn*-1 position, whereas at the *sn*-2 position they are generally enriched with ester-linked polyunsaturated fatty acids (PUFA). Among the membrane phospholipids, Pls are considered essential for brain function. PlsE, which are present at high levels in differentiated cells, are abundant in the brain and is believed to be involved in neuronal protection^66^.

When using SH-SY5Y cells as a cellular model to study brain lipid metabolism, it should be considered that they share significant differences compared to the phospholipid composition of the brain^67^. Several studies in human and mouse tissue have shown that CL fatty acid composition is highly tissue specific^68,69^. In particular, the brain has a unique and diverse acyl chain CL profile enriched in long-chain FA (i.e., C20:4 and C22:6), in contrast to other mammalian tissues (e.g., heart, skeletal muscle, and liver) which have much more homogeneous acyl chain pattern defined by the preferential incorporation of linoleic acid (C18:2)^70^. Differentiated RA-FUdr-SH-SY5Y cells with a reduced content in CL species with saturated fatty acids (probably palmitate or stearate), in favor of CL species with long chain PUFA, are consistent with the lipid changes that occur during the differentiation process and with the brain-specific CL profile. In this context, it has been reported that the acyl-CoA:lysophosphatidylglycerol acyltransferase 1, an important precursor for the CL synthesis^71^, is the most up-regulated protein identified by LC–MS/MS analysis in differentiated *versus* undifferentiated SH-SY5Y cells^72^.

Extensive metabolic reprogramming occurs during cellular differentiation^1,2,5,73–76^ and measuring metabolic profiles may help to determine whether differentiated SH-SY5Y cells can be used as a cellular model to study neurodegeneration. In this study, we observed higher basal and coupled respiration, in differentiated cells compared to undifferentiated cells, which are required to meet the high energy demands of mature neuronal cells. Likewise, we observed higher maximal mitochondrial respiratory activity and SRC, suggesting that differentiated cells are more capable of increasing their energy production on demand than undifferentiated cells. Conversely, the undifferentiated cells are very close to their maximal activity under basal conditions. Mitochondria activity is a highly dynamic process and cells can mobilize SRC when needed. This parameter therefore characterizes the mitochondrial ability to meet additional energy demands above the basal level, in response to acute cellular stressors such as Ca^2+^ overload, reactive oxygen, and nitrogen species (ROS/RNS) or heavy workload^77–80^, avoiding ATP crisis. It has been suggested that the cells with low SRC are generally proliferative cells that, likely, draw on the mitochondrial reserve to respond to biosynthetic needs for cell replication^81^. Conversely, high SRC levels characterize highly differentiated postmitotic cells like neurons^82^. It is well known that mature neurons are highly dependent on mitochondrial OXPHOS for their high energy requirements, especially to maintain neuronal excitability and general synaptic function of the brain^40,83–85^, which consumes about 20% of energy at rest, although it represents only around 2% of the total human body^86^.

Although there was no statistical difference in the basal rate of glycolysis between differentiated and undifferentiated cells, we observed a higher glycolytic capacity and reserve capacity in differentiated cells, suggesting that under physiologically energy demanding conditions, differentiated cells could rapidly increase their glycolytic function to further ensure ATP demand for their needs.

However, it should be noted that total extracellular acidification is a result of glycolytic lactate production and oxidative reactions. We observed a significant increase in the PPR associated with bicarbonate formed by CO_2_ production in differentiated cells compared to undifferentiated cells, as well as a higher OXPHOS-dependent ATP production rate as the dominant ATP generation pathway.

Overall, our results highlighted a different metabolic profile of differentiated cells compared to undifferentiated cells: the undifferentiated cells display a low energy profile, probably because the intermediates of glycolysis and TCA are used for biosynthetic rather than catabolic pathways to meet the anabolic needs of the cells. Differentiated cells, which no longer need to maintain a high replication rate and therefore have a lower anabolic demand, display an energetic metabolic profile, based mainly on OXPHOS activity, to support cellular homeostasis and their increasingly specialized functions, according to a variety of differentiation protocols^5,38,39,72,87^. It has already been described that increased SRC levels lead to a parallel increase in mitochondrial biogenesis^88^. PGC1α is the major regulator signalling pathways involved in mitochondrial biogenesis^89,90^. Activation of PGC1α-dependent biogenesis was observed in human melanoma with high levels of SRC^91^. Together with the reprogramming of mitochondrial bioenergetics, neuronal differentiation is associated with an increase in mitochondrial biogenesis^1,92^. We observed that mitochondrial transcription factors mainly involved in mitochondrial biogenesis, such as *NRF-1*, *PPARGC1A (PGC-1α)*, and *SIRT-1*, were strongly upregulated in differentiated cells compared with undifferentiated cells as well as mtDNA levels. Our results are consistent with previous reports showing that active mitochondrial biogenesis occurs during dendritic cell differentiation^93^ and osteogenic differentiation of human mesenchymal stem cells^94^.

The role of cAMP signalling in regulating OXPHOS activity and mitochondrial biogenesis has been widely described in a wide range of cell cultures^95,96^. In addition, several studies have shown that hormones and neurotransmitters involved in the upregulation of intracellular levels of cAMP can promote differentiation in neuronal cells^97,98^ and that differentiation of SH-SY5Y with dibutyryl cyclic AMP (dbcAMP) leads to neurite extension and increased expression of neuronal markers^99^. We observed that RA-FUdr-induced neuronal differentiation of SH-SY5Y was associated with an increase in cAMP levels and CREB phosphorylation. This agrees with the increased oxidative metabolism observed in differentiated cells. The increase in mitochondrial metabolism could also be related to a rearrangement of mitochondrial respiratory complexes into SCs, probably related to a different CL composition, as mentioned above. Indeed, several studies have shown that the balance between the individual mitochondrial respiratory complexes and the arrangement of SCs in the inner mitochondrial membrane is important to achieve the optimal performance of the electron transfer chain by shortening the distance between the respiratory complexes^100–102^. Moreover, recently, it has been reported the activation of the cAMP cascade leads to increased formation of respiratory SCs associated with higher OXPHOS capacity^54,103^. This prompted us to investigate the organization of the respiratory SCs in our cellular model. We observed a differential distribution of respiratory chain complex I and complex III between individual mitochondrial respiratory complexes and SCs, with an increased amount of SCs in differentiated cells, which could explain the increase in respiratory capacity. To our knowledge, this is the first time that the levels of respiratory SCs have been analysed in SH-SY5Y-differentiated cells. The increased levels of respiratory SCs in RA-FUdr-treated SH-SY5Y may be responsible for the increase in oxidative capacity, which plays a key role in changing the mitochondrial bioenergetic phenotype during differentiation into neuron-like cells. These results are consistent with reports showing that in neurons mitochondrial complex I is predominantly assembled into respiratory SCs, contributing to a higher respiratory rate, whereas in astrocytes, which exhibit glycolytic metabolism, a smaller proportion of complex I is assembled into respiratory SCs^104^. In addition, some authors have shown that adipogenic differentiation of hMSCs is accompanied by enhanced formation of respiratory SCs, which is associated with mitochondrial biogenesis and increased oxidative metabolism^105^.

In conclusion, in this study, we used a two-step differentiation protocol in which SH-SY5Y cells were treated with RA supplemented with the mitotic inhibitor FUdr to obtain a homogeneous functional population of neuron-like cells. Specifically, after RA-FUdr treatment, we observed a morphological neuronal phenotype in differentiated SH-S5Y5 confirmed by increased expression of neuron-specific markers, high levels of Pls species and neuron-like electrophysiological maturation. Furthermore, MALDI-TOF/MS analysis revealed significant changes in the acyl chain composition of CL, showing enrichment of long-chain and unsaturated FA. The short-term RA-FUdr-differentiation process resulted in the acquisition of high OXPHOS capacity, associated with a shift towards a mitochondrial energy phenotype, likely related to cAMP signalling pathway activation and respiratory SCs assembly.

## Methods

### Materials

All materials for the Extracellular Flux assays were from Seahorse Biosciences. Carbonyl cyanide p-[trifluoromethoxy]-phenyl-hydrazone (FCCP), oligomycin, rotenone, antimycin A and 2-Deoxy-d-glucose were from Sigma Aldrich. All lipid standards were purchased from Avanti Polar Lipids (Alabaster, AL, USA). 9-aminoacridine hemihydrate (9-AA) was used as a matrix for MALDI-TOF/MS analyses and was purchased from Acros Organics (Morris Plains, NJ, USA). All chemicals for the electrophysiology assays were from Sigma-Aldrich.

### Cell culture and differentiation

Human SH-SY5Y neuroblastoma cells were purchased from the American Type Culture Collection (ATCC, Manassas, Virginia, USA) and cultured in high-glucose Dulbecco’s modified Eagle’s medium (DMEM) supplemented with 10% (v/v) fetal bovine serum (FBS), 2 mM l-glutamine and 1% (v/v) penicillin/streptomycin, at 37 °C in a humidified atmosphere of 5% CO_2_. SH-SY5Y cells used for the experiments were controlled between passages 24 and 30. For the SH-SY5Y differentiation, cells were seeded at a density of 5 × 10^3^ cells/cm^2^ in 100 mm diameter culture dishes (Corning) and the treatment was performed essentially as described in^35,36^. After 48 h of plating, cells were cultured in fresh media supplemented with 1% FBS and 10 µM all-trans retinoic acid (RA, Sigma Aldrich) (dissolved in dimethyl sulfoxide, DMSO) for 5 days, and, for an additional 2 days, with RA in the presence of 10 µM 2-deoxy-5-fluorouridine (FUdr, Sigma Aldrich) (dissolved in water), to inhibit non-differentiated cells proliferation. The undifferentiated cells were treated with dimethyl sulfoxide (DMSO, vehicle) in a low serum medium for seven days and supplemented with FUdr for the last two days of treatment. All treatments were performed under dark conditions with medium change every 48 h. In this study, experimental groups were as follows: DMSO-FUdr-treated cells (undifferentiated cells) and RA-FUdr-treated cells (differentiated cells). The undifferentiated and differentiated cells were utilized the day after the seventh day of treatment for all the analysis performed.

### Immunofluorescence

For immunofluorescence staining, cells were fixed in 4% paraformaldehyde (PFA) in phosphate-buffered saline (PBS) for 20 min at room temperature. After washing in PBS for 15 min, the cells were permeabilized in 0.1% Triton X-100 in PBS for 20 min and washed 3 × for 10 min in PBS. After blocking non-specific binding sites, cells were incubated overnight with a primary antibody solution containing 0.5% bovine serum albumin (BSA), 0.1% Triton-X-100 in PBS, 5% fetal bovine serum and 0.02% NaN_3_. Cells were incubated in primary antibody against MAP2 (1:1000; Chemicon) at 4 °C overnight. Cells were washed several times in PBS before incubation for 1 h at room temperature with the appropriate Alexa-labeled secondary antibody (Alexa 594, Invitrogen). For nuclear staining, Fluoromount-G™ Mounting Medium (Invitrogen) containing DAPI was used. Images were acquired using the Cytation 5 Cell Imaging Multi-Mode Reader (BioTek) with a 20 × objective lens.

### Electrophysiology

Electrophysiological activities of SH-SY5Y cells were recorded by the tight-seal whole-cell recording at room temperature (20–23 °C). Coverslips were transferred to the recording chamber and continuously superfused with the recording solution at a rate of ~ 2 mL/min. Signals were recorded using HEKA EPC10 patch-clamp amplifier (HEKA Elektronik). Data acquisition was made with Patchmaster next 1.2. All signals were low-pass filtered at 2.9 kHz (Bessel filter), and data analysis was performed using R Studio (version 2022.02.3). The sampling frequency was 100 kHz. Patch electrodes were pulled from borosilicate glass pipettes (Warner Instruments) with 2–3.5 MΩ resistance and filled with the intracellular solution containing (mM): 4 NaCl, 140 KCl, 2 MgCl2, 10 HEPES, 0.8 EGTA (pH 7.2 adjusted with KOH). The extracellular solution contained (mM): 125 NaCl, 4 KCl, 1 MgCl_2_, 2 CaCl_2_, 10 HEPES, and 10 glucose (pH 7.4 adjusted with NaOH). Experiments were performed on relatively smaller cells to achieve better voltage clamp and to have lower membrane capacitance. Cells were held at − 70 mV and depolarizing pulses were applied in 10 steps of 100 ms, with an increment of 10 mV each. After the whole-cell configuration was obtained, recordings were repeated at least 3 times from each cell for averaging the recorded current responses. The resting membrane potential was measured in c-clamp mode.

### Lipidic extraction and analysis by MALDI-TOF/MS

Cells were harvested from Petri dishes with 0.05% trypsin and 0.02% EDTA, pelleted by centrifugation at 500×*g* and then resuspended in cold phosphate-buffered saline at 4 °C, supplemented with proteinase inhibitor (IP). Total lipids were extracted by a modified Bligh & Dyer method^106^. Briefly, cell aliquots (about 200 μg of proteins) were diluted up to 450 μL with distilled water; then 500 μL methanol and 500 μL chloroform were added to the aqueous phase and shaken for 30 min; after centrifugation, the lower organic phase was collected, carefully dried under N2 stream and then dissolved in 10 μL of chloroform. The extraction procedure including sample preparation was performed at 4 °C to prevent lipid degradation. The total lipid extract was mixed with 10 μL of the matrix solution (10 mg/mL 9-AA in 2-propanol/acetonitrile 60/40 v/v). Then, 0.35 μL of the mixture was spotted in triplicate on the MALDI target to be analyzed. In the present study, we have analyzed lipids extracted from three different preparations for undifferentiated and differentiated cells. MALDI-TOF mass spectra of total lipid extracts were acquired in negative and positive ion modes on a Bruker Microflex LRF mass spectrometer (Bruker Daltonics, Bremen, Germany). The system used a pulsed nitrogen laser (337 nm) and the extraction voltage was 20 kV. For each mass spectrum, 2000 single laser shots (sum of 4 × 500) were averaged. The laser fluence was kept about 5% above the threshold to a good signal-to-noise ratio. The delayed pulsed extraction acquired all spectra in a reflector mode (detection range: 400–2000 mass/charge, m/z). A mixture of lipid standards was spotted next to the sample, and external calibration was performed before each measurement, as previously described^107,108^. Peak areas, spectral mass resolutions, and signal-to-noise ratios were determined using Flex Analysis 3.3 (Bruker Daltonics Bremen, Germany) software.

### Metabolic flux analysis

Real-time bioenergetic parameters, Oxygen Consumption Rate (OCR) and Extracellular Acidification Rate (ECAR) were measured in adherent cells by an XF24 Extracellular Flux Analyzer (Seahorse Bioscience, Billerica, MA, USA). After optimization of the seeding density, cells at 10 × 10^3^ cells/well on XF24 cell culture microplates were treated for 7 days as previously described. The day after the seventh day of treatment, cells were pre-incubated for at least 1 h at 37 °C in a non-CO_2_ incubator in XF assay media (pH 7.4, Seahorse Biosciences) supplemented with pre-warmed 10 mM glucose, 2 mM l-glutamine and 1 mM sodium pyruvate. The XF Cell Mito Stress Test (Seahorse Bioscience) was used to measure the key parameters of mitochondrial respiration using specific mitochondrial inhibitors and uncouplers. Specifically, for OCR analysis, after optimization of the concentrations to elicit maximal effects, oligomycin (1 µM), carbonylcyanide ptrifluoromethoxyphenylhydrazone (FCCP) (1 µM), rotenone + antimycin A (1 µM + 1 µM), were sequentially injected, according to the manufacturer’s instructions, into each well after basal measurements to evaluate the ATP-linked respiration, the maximal respiratory capacity, and the non-mitochondrial oxygen consumption, respectively. For ECAR analysis, at the end of running, 100 mM 2-deoxyglucose was added to determine glycolysis-independent acidification. Oxygen consumption rate (OCR) for mitochondrial respiration and extracellular acidification rate (ECAR) were measured for 3 min with 3 min of mixing and 2 min of the waiting period. Each experimental point is an average of five replicate wells and each experiment was performed at least three times with different plates. The OCR and ECAR values were normalized to protein content in each well, determined by the Pierce BCA Protein Assay Kit (Thermo Fisher Scientific). The cellular ATP production rates were quantified using the Agilent Seahorse XF ATP real-time assay kit using label-free technology in real time as specified by the manufacturer and essentially as described in^51,53^. According to the equation: glucose + 2 ADP + 2 Pi → 2 lactate + 2 ATP + 2 H_2_O + 2 H^+^, the glycolytic ATP production rate (GlycoATP) was estimated from glycolytic proton production rate (glycoPPR) after determination of the buffering power (BP). BP was determined separately by measuring the pH changes elicited by successive additions of titrated HCl in XF assay media in the presence of the same number of assayed cells and was found to be 3.1 mmol H^+^/L/pH. The mitochondrial ATP production rate (MitoATP) was equal to OCR_ATP_ × 2 × P/O (mol ATP/mol O), where OCR_ATP_ was the difference between basal OCR and OCR in the presence of oligomycin. A theoretical P/O ratio of 2.25 was assumed, based on the combined oxidation of glucose, fatty acid, and glutamine.

### RNA isolation reverse transcription and quantitative PCR

The purification of total RNA from cells was carried out by using Aurum Total RNAMini Kit (Bio-Rad, Hercules, CA, USA) according to the manufacturer’s protocol. The control sample was represented by undifferentiated cells. Complementary DNA (cDNA) was synthesized by reverse-transcription of total RNA using the iScript cDNA Synthesis kit (Bio-Rad, Hercules, CA, USA), following the manufacturer’s instructions. Semi-quantitative determination of mRNA levels was performed by Real-Time Quantitative Reverse Transcription PCR (qRT-PCR) using SsoAdvanced Universal SYBR® Green Supermix (Bio-Rad, Hercules, CA, USA). Reactions were performed in a CFX96 Touch Real-Time PCR Detection System (Bio-Rad Laboratories, Hercules, CA, USA) in duplicate for each sample for three independent experiments. Relative quantification was performed using the comparative CT method (ΔΔCT). Quantitative normalization for each sample was performed by using glyceraldehyde-3-phosphate dehydrogenase (*GAPDH*) as an internal control. Validated primers for semi-qRT-PCR are provided in Table 2. Mitochondrial DNA quantification was assessed essentially as previously described^109^. Total DNA was extracted from undifferentiated and differentiated cells using the Wizard® Genomic DNA Purification Kit (Promega) according to the manufacturer’s instructions. *mt-ND1*, as mtDNA target, and 18 S as a reference for the nuclear DNA content, were amplified by quantitative Real-Time PCR in a CFX96 Touch Real-Time PCR Detection System (Bio-Rad Laboratories, Hercules, CA, USA) using SsoAdvanced Universal SYBR® Green Supermix (Bio-Rad, Hercules, CA, USA). The difference in threshold cycle values ΔCt was used as a measure of the relative abundance of the mitochondrial genome. In particular, the mtDNA/nDNA ratio is reported as 2^-ΔCt^. Validated primers for q-PCR are reported in Table 2.

**Table 2:** Oligonucleotide sequences for Real-time PCR.

	*FORWARD*	*REVERSE*
*MAP2*	5′-ATTGACAGCCAAAAGTTGAA-3′	5′-TCGAGCAGGTTGATGCTTCC-3′
*TUBB3*	5′-CTCAGGGGCCTTTGGACATC-3′	5′-CAGGCAGTCGCAGTTTTCAC-3′
*TH*	5′-GCAGTTCTCGCAGGACATTG-3′	5′-TCTGCTTACACAGCCCGAAC-3′
*ENO2*	5′-ATCCTTCCCGATACATCACTGG-3'	5′-TGGTCAAATGGGTCCTCAAT-3′
*PPARGC1A*	5′-AAACAGCAGCAGAGACAAATGC-3′	5′-TTGGTTTGGCTTGTAAGTGTTGTG-3′
*SIRT1*	5′-TGGCAAAGGAGCAGATTAGTAGG-3′	5′-CTGCCACAAGAACTAGAGGATAAGA-3′
*NRF1*	5′-CCGGAAGAGGCAACAAACAC-3′	5′-TGTCCCACACGAGTAGTATATTCATCT-3′
*TFAM*	5′-AAGGAAAACTGGAAAAATCTGTCTG-3′	5′-CGTCCAACTTCAATCATTTGTTCT-3′
*SREBP-1C*	5'-ACACCATGGGGAAGCACAC-3′	5′-CTTCACTCTCAATGCGCC-3′
*FASN*	5′-GAAGGAGGGTGTGTTTGCC-3′	5′-GGATAGAGGTGCTGAGCC-3′
*GAPDH*	5′-CAACTTTGGTATCGTGGAAGGAC-3′	5′-ACAGTCTTCTGGGTGGCAGTG-3′
*mt-ND1*	5′-CCTTCGCTGACGCCATAAA-3′	5′-CGGTGATGTAGAGGGTGATG-3′
*18S*	5′-GGCGTTATTCCCATGACC-3′	5′-AACCATCCAATCGGTAGTAGC-3′

### Cyclic adenosine monophosphate (cAMP) assay

cAMP assay was assessed essentially as previously described^54^. Following RA treatment, cells were incubated with 400 µL of 0.1 M HCl containing 0.1% triton X-100 for 10 min at 37 °C, lysed by manual scrapping and centrifuged at 1300×*g* for 10 min at 4 °C. cAMP levels in clarified lysates were measured using the Direct cAMP ELISA Kit (Enzo Life Sciences, New York, NY, USA) according to the manufacturer’s protocol. Absorbance was measured on a Victor 2030 multilabel reader (PerkinElmer, Waltham, MA, USA). The cAMP values were normalized to the protein concentration and expressed as pmol/mg protein.

### Sample preparation, electrophoretic procedures and Western blot analysis

Sodium-Dodecyl-Sulphate Polyacrylamide Gel Electrophoresis (SDS-PAGE) was performed according to Schägger et al.^110^. Cells were resuspended in hypotonic buffer supplemented with an antiproteases cocktail tablet (Roche, Basel, Switzerland) and freeze-thawed three times. 35 µg of total proteins, quantified by the Bradford protein assay (Bio-Rad, Milan, Italy), were resolved on a 12% SDS-PAGE and transferred to a nitrocellulose membrane using a Trans-Blot Turbo Transfer System (Bio-Rad, Milan, Italy). Ponceau S staining was used to demonstrate equal loading. Western blot analysis was performed using the specified primary antibodies against CI-NDUFA9 (1:1000; Thermo Fisher-Invitrogen,), CIII-Core 2 subunit (1:1000; Thermo Fisher-Invitrogen), β-ACTIN (1:8000; Sigma Aldrich), GAPDH (1:3000; Santa Cruz), CREB (1:1000; Cell Signaling) and phopho-CREB (1:1000; Cell Signaling). After 4 °C overnight incubation, the membranes were further probed at 4 °C for 1 h with a correspondingly suited horseradish peroxidase-conjugated secondary antibody (1:10,000; Bio-Rad, Milan, Italy). The proteins were detected by chemiluminescence (Pierce™ ECL Western Blotting Substrate, Thermo Scientific, Rockford, IL, USA) and immunoreactive bands were quantified using Image Lab Software (BioRad Laboratories Inc., Hercules, CA, USA) and normalized against β-actin expression (1:8000; Sigma-Aldrich).

Respiratory chain complexes and SCs were separated by first-dimension (1D) blue-native electrophoresis (BNE). Whole-cell lysates (150 µg) were suspended in 750 mM aminocaproic acid, 50 mM Bis–Tris, 0.5 mM EDTA (mitochondrial buffer), plus 4:1 (mg/mg) digitonin. After 10 min on ice, the samples were centrifuged at 20,000×*g* for 30 min and the supernatant was recovered and kept on ice. The supernatants, representing the mitochondrial solubilized proteins, were loaded on 3–13% gradient native gel followed by immunoblotting with antibodies against NDUFS1 subunit of complex I (1:250 dilution, Santa Cruz Biotechnology, Dallas, TX, USA) and Core II subunit of complex III (1:1000 dilution, Thermo Fisher–Invitrogen).

### Statistical analysis

Data are reported as mean ± standard error mean (SEM) of at least three independent experiments. For each data set, the Shapiro–Wilk normality test was applied to determine if the data had a normal distribution. Significant differences were determined using the Student’s t-test or Welch and Brown-Forsythe versions of one-way ANOVA when the dependent variable was normally distributed and the Mann–Whitney test when the distribution did not fit the normality assumption. The threshold for statistical significance was set to 0.05. All analyses were performed using Graph Pad Prism Software Version 8 (Graph Pad Software, San Diego, CA, USA).

### Supplementary Information

Supplementary Information.

## Data Availability

All extracted data is available in the paper and supplementary materials. Further information is available on request.
