# Supplementary material for: High OXPHOS efficiency in RA-FUdr-differentiated SH-SY5Y cells: involvement of cAMP signalling and respiratory supercomplexes
Source: Sci Rep. 2024 Mar 28;14:7411. doi: 10.1038/s41598-024-57613-x (PMC10978939; doi:10.1038/s41598-024-57613-x)
Supplement: Supplementary file 1 — Supplementary Information. [file 41598_2024_57613_MOESM1_ESM.pdf]

<u>High OXPHOS efficiency in RA-FUdr-differentiated SH-SY5Y cells: involvement of cAMP signalling and respiratory supercomplexes</u>

Maria Laura Matrella[1], Alessio Valletti[1,5], Isabella Gigante[2], Domenico De Rasmo[3], Anna Signorile[1], Silvia Russo[1], Simona Lobasso[1], Donatella Lobraico[1], Michele Dibattista[1], Consiglia Pacelli [4,*,+] and Tiziana Cocco[1,*,+]

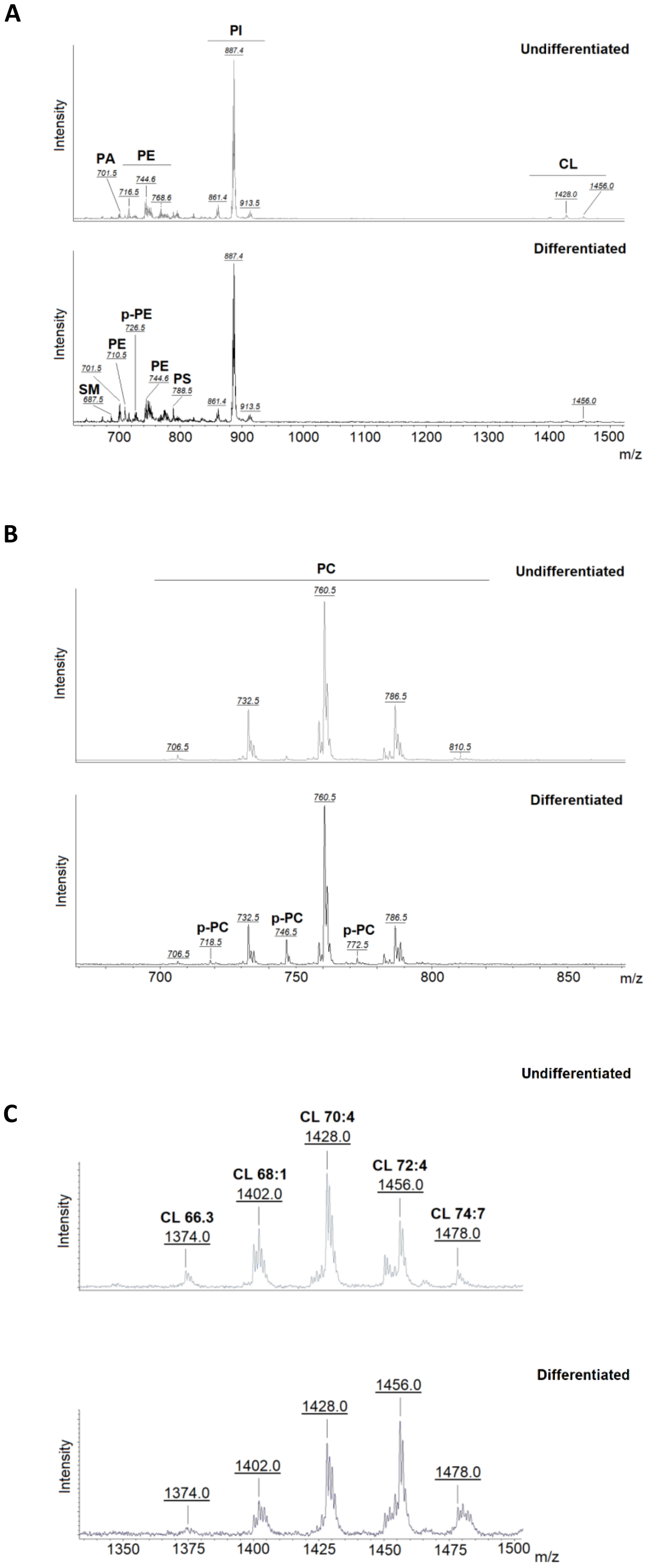

Figure S1. Comparative MALDI-TOF/MS lipid profiles between undifferentiated and RA-Fudr-differentiated cells. Typical mass spectra obtained in negative **(A)** or positive **(B)** ion mode. **(C)** A comparison of cardiolipin fingerprinting obtained by MALDI analysis in negative ion mode. Lipid assignments for the main signals are indicated, as in Table S1.

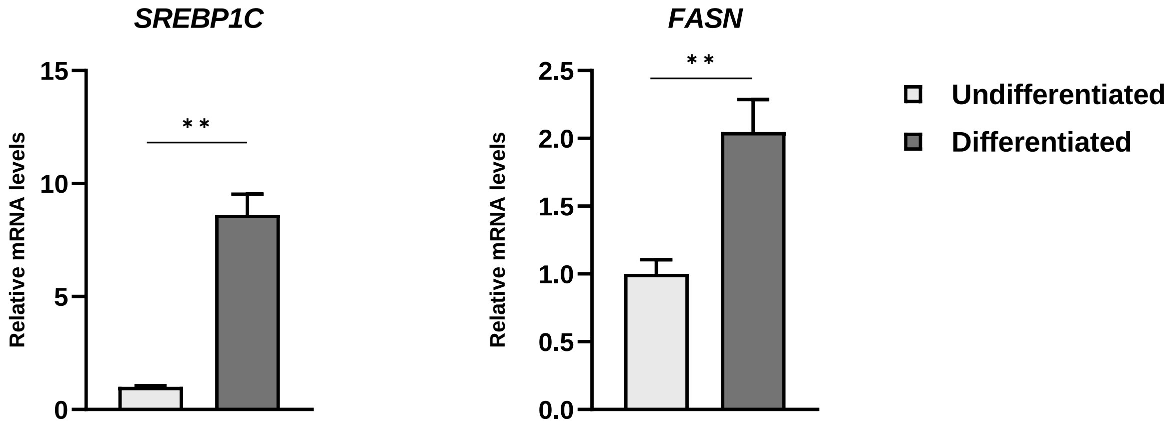

Figure S2. Transcription levels of *SREBP1C* and *FASN*, genes involved in lipid metabolism. Relative mRNA levels were evaluated by qRT-PCR and normalized to the housekeeping gene *GAPDH*. Data, expressed as fold-changes mRNA expression levels in differentiated cells, compared to undifferentiated cells, are means of two replicates ± SEM from three independent experiments. Statistical analyses were performed using unpaired t-test with Welch's correction (***p* < 0.01).

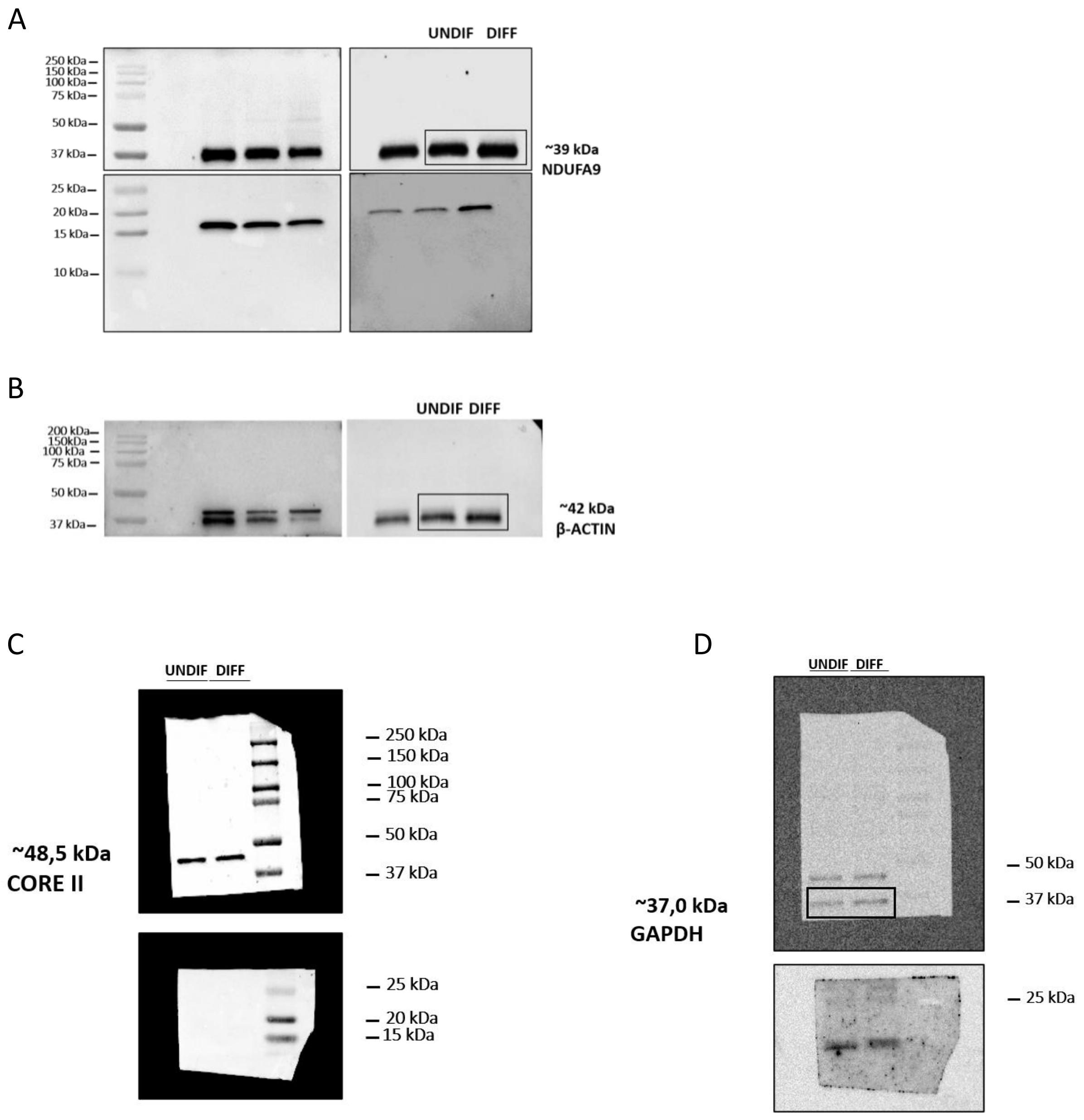

Figure S3. The uncropped images for Figure S4. Whole-image Western blot for NDUFA9 **(A)**. Notably, the bands corresponding to the 37 kDa ladder, as indicated by the rectangle, were analyzed. There were bands corresponding to other samples. **(B)** Whole-image Western blot for β-ACTIN used as a loading control. Specifically, the bands just above the 37 kDa ladder were analyzed, as indicated by the rectangle. **(C)** Whole-image Western blot for CORE II. Notably, the bands above the 37 kDa ladder were analyzed. **(D)** Whole-image Western blot for GAPDH used as a loading control. Specifically, the bands corresponding to the 37 kDa ladder were analyzed, as indicated by the rectangle.

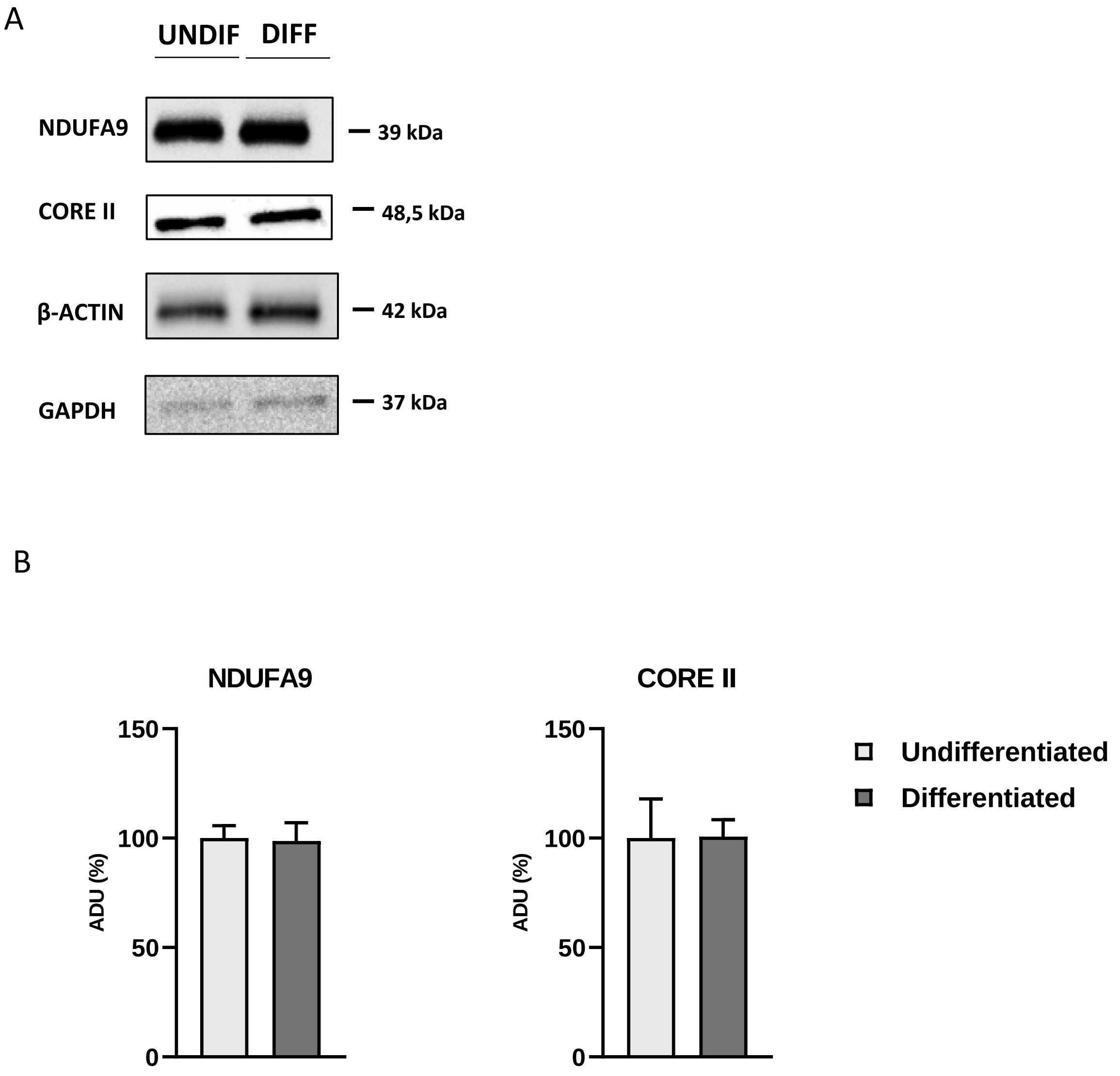

Figure S4. Western blot analysis of the expression of NDUFA9 and CORE II proteins. (**A**) Representative Western blot of NDUFA9 (complex I) and CORE II (complex III) performed on whole cell lysate of undifferentiated and differentiated cells. Proteins were loaded on 12% SDS-polyacrylamide gel electrophoresis (PAGE). After separation, the proteins were transferred to nitrocellulose membranes and immunoblotted with the antibodies against NDUFA9 and CORE II. (**B**) Bar graph shows quantification by densitometric analysis of mitochondrial protein bands normalized to β-actin and GAPDH used as loading controls. Data of arbitrary densitometric units (ADU) are means±SEM of three independent experiments and expressed as a percentage of undifferentiated cell values.

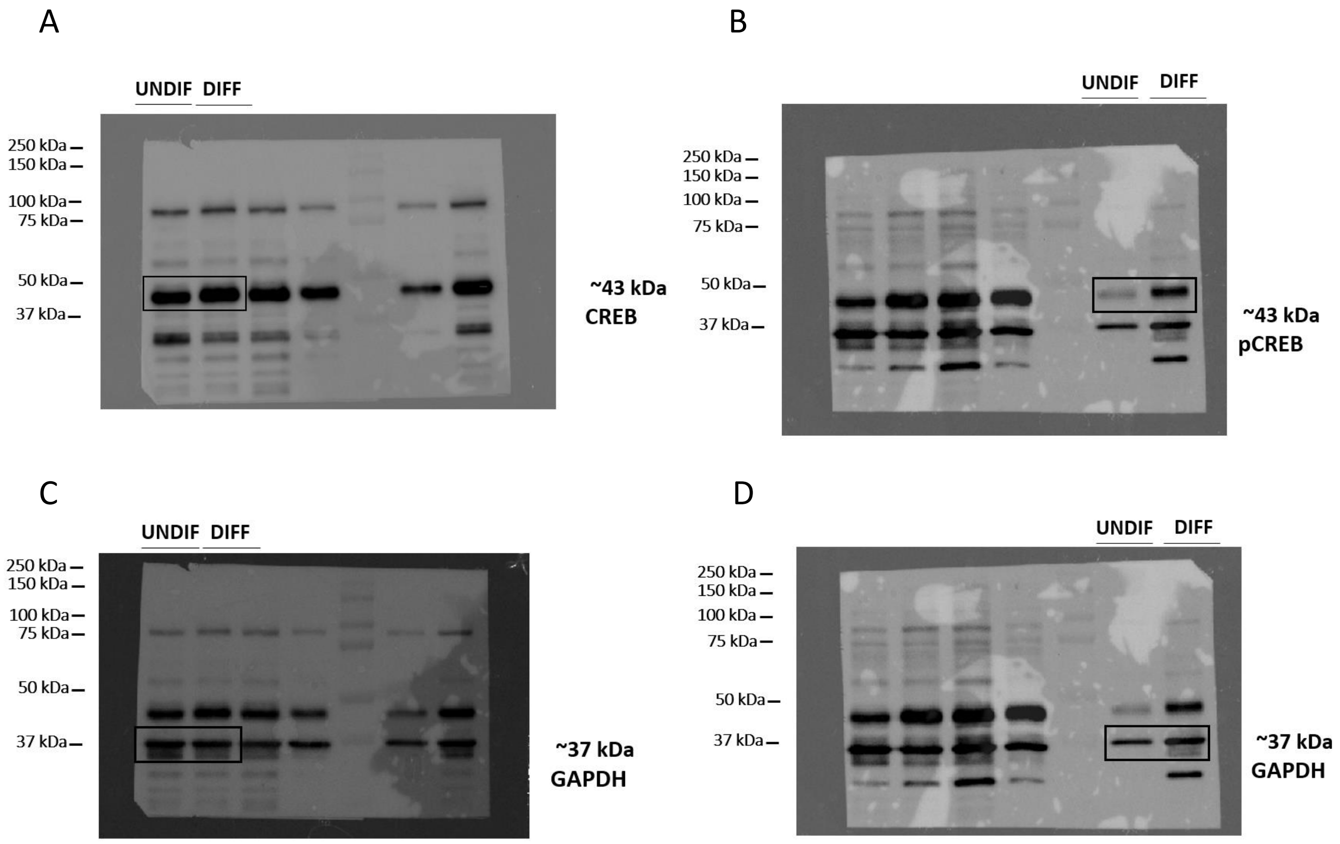

Figure S5. The uncropped images for Figure 6B. Whole-gel Western blot for CREB **(A)** and p-CREB (B). Notably, the bands just above the 37 kDa ladder, as indicated by the rectangle, were analyzed. There were bands corresponding to other samples. **(B)** Whole-gel Western blot for GAPDH **(C-D)** used as loading control. In particular, the bands corresponding to the 37 kDa ladder were analyzed, as indicated by the rectangle. There were bands corresponding to other samples.

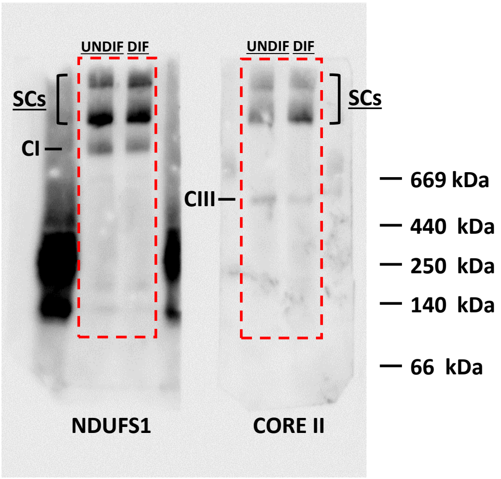

Figure S6. Original images of full-length blots shown in Figure 7A. Full-length blot for NDUFS1 and COREII**.** Cropped areas shown in Figure 7A are marked in red colour.

m/z value	[M-H]$^-$	[M+H]$^+$
647.4	PA 32:0	
673.4	PA 34:1	
687.5	SM 16:0	
701.5	PA 36:1	
706.5		PC (30:0)
710.5	PE 34:4	
716.5	PE 34:1	
718.5		PlsC(P-32:0)
726.5	PlsE (P-36:2)	
732.5		PC (32:1)
744.6	PE (36:1)	
746.6		PlsC(P-34:0)
747.6	PG (34:1)	
758.6		PC (34:2)
760.6		PC (34:1)
762.5	PE (38:6)	
768.5	PE (38:3)	PlsC (P-36:3)
772.5		PlsC (P-36:1)
774.5	PE (38:0)/PlsS (P-36:0) /PlsE (P-40:6)	
782.5		PC (36:4)
786.5		PC (36:2)
788.5	PS (36:1)	PC (36:1)
796.5	PE (40:4)	PlsC (P-38:3)
810.5		PC (38:4)
812.4	PS (38:3)	PC (38:3)
834.4	PS (40:6)	
861.4	PI (36:2)	
885.4	PI (38:4)	
887.4	PI (38:3)	
913.5	PI (40:4)	
1,374.1	CL (66:3)	
1,402.1	CL (68:1)	
1,428.1	CL (70:4)	
1,456.1	CL (72:4)	
1,478.2	CL (74:7)	

Table S1. Lipid assignments of m/z values detected in negative and positive ion mode MALDI-TOF mass spectra of undifferentiated and RA-Fudr-differentiated cells. The numbers (a:b) define the total length (as carbon numbers) and number of double bonds of acyl chains, respectively.

CL= cardiolipin; PA = phosphatidic acid; PC = phosphatidylcholine; PE = phosphatidylethanolamine; PG = phosphatidylglycerol; PI = phosphatidylinositol; Pls = plasmalogen; PS = phosphatidylserine; SM = sphingomyelin.
